# Global analysis of the MATE gene family of metabolite transporters in tomato

**DOI:** 10.1186/s12870-017-1115-2

**Published:** 2017-10-30

**Authors:** Adolfo Luís dos Santos, Samuel Chaves-Silva, Lina Yang, Lucas Gontijo Silva Maia, Antonio Chalfun-Júnior, Senjuti Sinharoy, Jian Zhao, Vagner Augusto Benedito

**Affiliations:** 1 Division of Plant and Soil Sciences, West Virginia University, 3425 New Agricultural Sciences Building, Morgantown, WV 26506-6108 USA; 2 Plant Molecular Physiology Laboratory, Biology Department, Federal University of Lavras (UFLA), Lavras, MG Brazil; 3 Department of Biotechnology, University of Calcutta, Kolkata, India; 4 State Key Laboratory of Tea Plant Biology and Utilization, College of Tea and Food Sciences, Anhui Agricultural University, Hefei, 230036 China

**Keywords:** Antiporter, Efflux, Genome evolution, Metabolic gene cluster, Regulatory gene network

## Abstract

**Background:**

Species in the Solanaceae family are known for producing plethora of specialized metabolites. In addition to biosynthesis pathways, a full comprehension of secondary metabolism must also take into account the transport and subcellular compartmentalization of substances. Here, we examined the MATE (Multidrug and Toxic Compound Extrusion, or Multi-Antimicrobial Extrusion) gene family in the tomato (*Solanum lycopersicum*) genome with the objective of better understanding the transport of secondary metabolites in this model species. MATE membrane effluxers encompass an ancient gene family of secondary transporters present in all kingdoms of life, but with a remarkable expansion in plants. They mediate the transport of primary and secondary metabolites using the proton motive force through several membrane systems of the cell.

**Results:**

We identified 67 genes coding for MATE transporters in the tomato genome, 33 of which are expressed constitutively whereas 34 are expressed in specific cell types or environmental conditions. Synteny analyses revealed bona fide paralogs and Arabidopsis orthologs. Co-expression analysis between MATE and regulatory genes revealed 78 positive and 8 negative strong associations (ρ≥|0.8|). We found no evidence of MATE transporters belonging to known metabolic gene clusters in tomato.

**Conclusions:**

Altogether, our expression data, phylogenetic analyses, and synteny study provide strong evidence of functional homologies between MATE genes of tomato and *Arabidopsis thaliana*. Our co-expression study revealed potential transcriptional regulators of MATE genes that warrant further investigation. This work sets the stage for genome-wide functional analyses of MATE transporters in tomato and other Solanaceae species of economic relevance.

**Electronic supplementary material:**

The online version of this article (10.1186/s12870-017-1115-2) contains supplementary material, which is available to authorized users.

## Background

The Solanaceae (nightshades) is a botanical family with globally important crops within about 90 genera and ~3000–4000 species, such as tomato, potato, eggplant, tobacco, petunia, chilies, and peppers. This family also contains crop species of more local relevance, such as tomatillo, goji, and gooseberry, not to mention countless medicinal, ornamental, toxic, and weed species [1]. Species within this family are known for their prolificacy in producing diverse secondary metabolites, especially alkaloids, and also phenolics and terpenoids [2–5]. Given the economic significance as a crop as well as its phenotypic and genetic advantages, the tomato (*Solanum lycopersicum*) has been chosen as the biological model species not only for this family, but also for the whole Asterid clade, which comprises of numerous agricultural species, including crops from varied families that produce relevant secondary metabolites, such as stimulant alkaloids in coffee (Rubiaceae), tea (Theaceae), and yerba mate (Aquifoliaceae). This clade also contains important Asteraceae crops (lettuce, sunflower, artichoke, stevia, echinacea, and daisies), and common Lamiaceae herbs (basil, lavender, marjoram, mint, oregano, rosemary, sage, thyme), to name just a few.

Moreover, the choice of the tomato over the well-established Rosid model, *Arabidopsis thaliana*, is justified not only by the closer relationship of tomato to other Asteridae crops, but also because many features of agricultural relevance are not available in Arabidopsis, such as development of a complex leaf pattern, climacteric fleshy fruit (as a botanical berry), establishment of symbiotic root interactions (e.g., mycorrhization) [6], as well as its abundant metabolism of specialized compounds, including alkaloids (e.g., tomatine) [7], phenolics (e.g., rutin, naringenin, apigenin, caffeic acid) [8], and terpenoids (including volatile components of the fruit aroma, such as geranial and norisoprenes) [9, 10].

The availability of the tomato genome sequence and other genetic resources (e.g., molecular markers and genetic maps, germplasm collection, and transcriptional data) allows for a global and focused analysis of gene functions in order to better understand the developmental and metabolic mechanisms with the ultimate goal of generating breeding toolkits to improve traits of agricultural relevance [11, 12]. For example, the manipulation of alkaloid transport in the Solanaceae may be key to producing solanine-free potatoes, generating tomato lines with increased levels of the beneficial glycoalkaloid tomatine, or even the domestication of poisonous wild species for food or feed [13].

MATE (Multidrug and Toxic Compound Extrusion or Multi-Antimicrobial Extrusion) transporters comprise a universal gene family of membrane effluxers present in all kingdoms of life. However, possibly due to the abundance of specialized metabolites characteristic of plant species, this family has vastly expanded in plant genomes. MATE proteins have typically 400–550 amino acid residues encompassing 12 transmembrane domains (TMD). However, this family lacks an absolute conservation of amino acid residue in its core domain sequence (IPR002528/PF01554) [14], which may allow for its great substrate diversity. Most MATE transporters export primary and secondary metabolites out of the cytosol using electrochemical gradient across the membrane [15], thus mediating the efflux or subcellular compartmentalization of metabolites in the cell (www.tcdb.org) [16]. Given the prominent roles in cell detoxification, MATE transporters in Arabidopsis are alternatively called DETOXIFICATION (DTX) proteins [17]. In plants, MATE transporters have been implicated directly or indirectly in mechanisms of detoxification of noxious compounds or heavy metals [17–19], tolerance to aluminum toxicity [15, 20–22], disease resistance [23, 24], nutrient homeostasis, such as Fe^3+^ uptake [21, 22, 25], and the transport of diverse types of secondary metabolites, such as alkaloids [26], flavonoids [27, 28] and anthocyanidins [29, 30], as well as hormones, such as ABA, salicylic acid, and auxin [31–33].

Previous research has characterized the functions of the MATE transporters in many species, such as bacteria, yeast, animals and plants [34–39]. Unlike mammalian genomes, which carries only a few MATE genes (e.g., 5 in mouse, 11 in human), plant genomes encode a large number of MATE genes: 56 in Arabidopsis [33], 45 in rice [40], 70 in *Medicago truncatula* [22] and 117 in soybean [34]. So far, soybean is the species with the highest number of MATE genes, which can be explained by the high rate of gene duplication of its paleopolyploid genome: 82% of the MATE genes are present in duplicate (thus potentially carrying redundant functions), being 21% arranged in tandem and 61% in large-scale segmental duplications [15]. Since MATE transporters carry essential functions in physiological mechanisms in plants, they could be ideal targets of breeding programs for improving traits of agricultural relevance, such as aluminum tolerance, iron nutrition, and accumulation of secondary metabolites of interest (e.g., increase of anthocyanin contents or eradication of toxic alkaloids). A complete analysis of the MATE gene family in a plant species is essential to fully comprehend its secondary metabolism. Among the many plant MATE transporters characterized to date, surprisingly none has been fully functionally characterized in tomato. Herein, aiming to guide future molecular studies, we identified 67 genes coding for MATE transporters in the tomato genome, and produced a genomic inventory of MATE genes in order to provide a close look into the functional roles MATE transporters may play in the tomato’s physiology and cellular metabolism. Co-expression analysis between each MATE and 3169 regulatory genes revealed 78 positive and 8 negative strong associations involving 12 MATE transporters. Importantly, no evidence of MATE transporters in known metabolic gene clusters was found in the tomato genome. This work sets the stage for further functional characterization of these MATE transporters as well as manipulation of traits in relation to tomato metabolism.

## Results and discussion

### Phylogenetic analysis of the MATE gene family in tomato

**Fig. 1:**
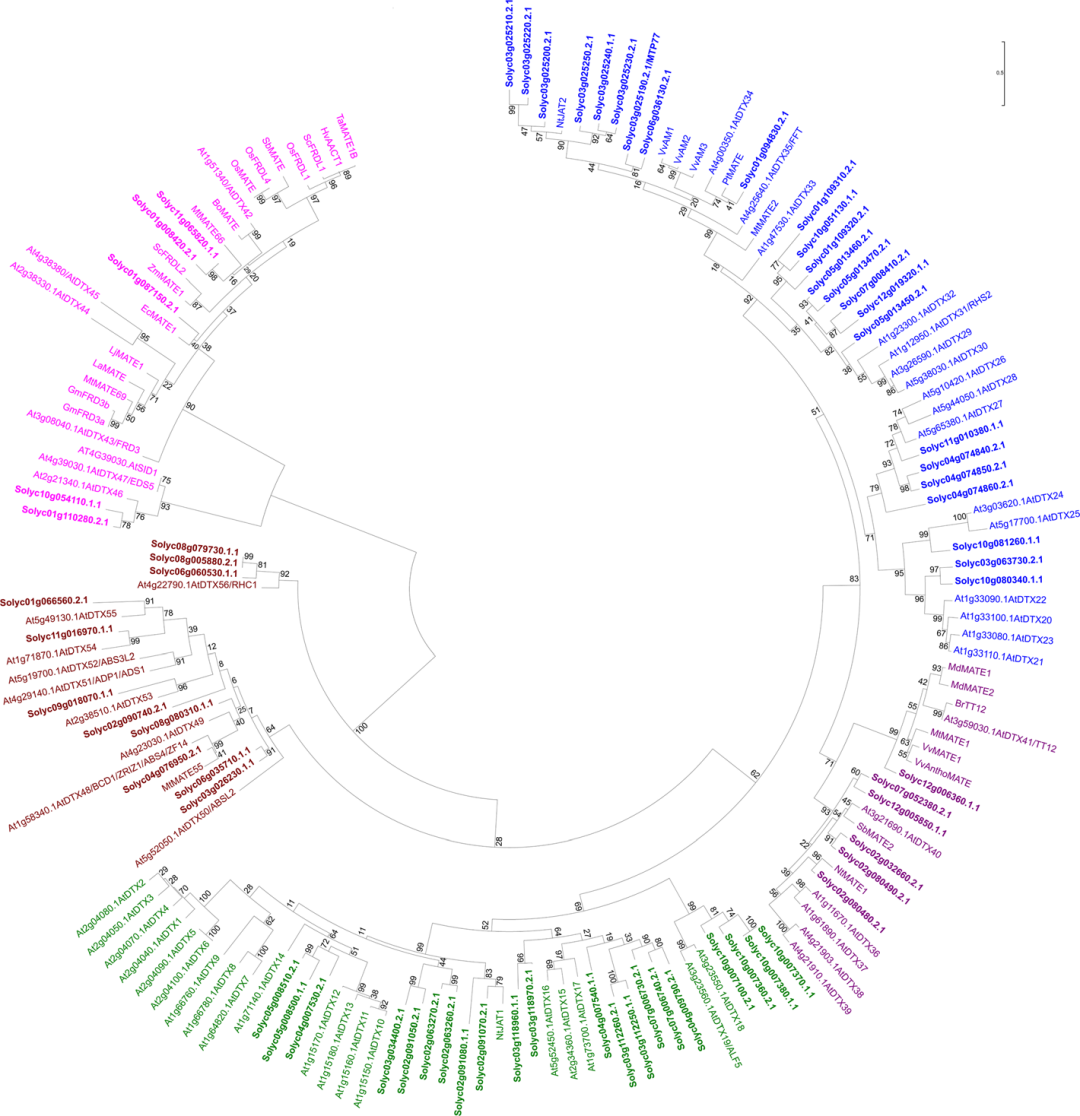
Phylogeny of MATE transporters in the tomato genome. All protein sequences of MATE transporters identified in the tomato genome (67 sequences), along the complete set in *Arabidopsis thaliana* (56 sequences) and other MATE transporters functionally characterized in other plant species (33 sequences). The analysis was conducted in MEGA7 [90] using Maximum Likelihood method with 1000 bootstraps. Branches are drawn to scale in the number of substitutions per site. Notice the five clear clades encompassing MATE with distinct functional properties

We identified 67 members of the MATE family of membrane transporters in *S. lycopersicum* (Additional file 1 Tables S1**,** Additional file 2 Table S2) by using a previously described analysis pipeline [41] and the TransportTP tool [http://bioinfo3.noble.org/transporter] [42]. Phylogenetic analyses of membrane transporters are usually not accurate to assign specific substrates. However, phylogeny of the MATE family has been shown to be quite useful to predict affinities with potential molecule groups, such as organic acids (e.g., citrate), flavonoids (anthocyanin, proanthocyanidin), and alkaloids (nicotine). Therefore, a phylogenetic analysis was performed with the 67 MATE protein sequences identified in tomato along with 56 from Arabidopsis as well as the other 33 MATE transporters that were functionally characterized in diverse plant species (Fig. 1).

The tree pattern obtained with five clades for the transport of potentially distinct substrates is congruent with previous studies [15, 43]. Clade 1 (blue) contains 24 tomato MATE transporters along with ROOT HAIR-SPECIFIC 2 (RHS2/DTX31) and FLOWER FLAVONOID TRANSPORTER (FFT/DTX35) from Arabidopsis; anthoMATE 1 to 3 (VvAM1 to VvAM3) from grapevine; PtMATE from poplar; and MtMATE2 from *Medicago truncatula*. The previously reported MTP77 (Solyc03g025190) from tomato cv. Micro-Tom [30] and JASMONATE-INDUCIBLE ALKALOID TRANSPORTER 2 (NtJAT2) from tobacco [44] are also confined to clade 1. Many members of this clade have been functionally implicated in the transport of secondary metabolites. Importantly, FFT/DTX35 was considered as flavonoid transporter [45], but recent findings showed that, along with DTX33, it rather functions as a vacuolar chloride channel involved in cell turgescence during stomatal movements [46], root hair elongation, and pollen germination. MtMATE2 is a vacuolar anthocyanin transporter [47]; VvAM1 and VvAM3 are involved in anthocyanin transport to vacuoles in the grapevine [48]; NtJAT2 transports alkaloids, such as nicotine, into the vacuole of the tobacco leaf cells [44]. The substrate of the root-specific RHS2 [49] remains unknown. In tomato, since MTP77 is induced by the MYB transcription factor ANTHOCYANIN 1 (ANT1/MYB113), it was presumed to be a transporter that directs anthocyanins to the vacuole of leaf cells [30]. Although its full functional characterization is still lacking, but given the recent characterization of Arabidopsis DTX33 and DTX35 function [46], MTP77 might rather work as a vacuolar chloride channel. Closely related to DTX24 and DTX25 from Arabidopsis, Solyc10g081260 has also been indirectly linked with phenolics transport for being induced in high-phenolics introgression lines of tomato and transcriptional repression in low-phenolics mutant lines compared to the wild type [8]. Generally, the tomato MATEs in this clade are excellent candidates for mediating transport and cellular accumulation of alkaloids and phenolic compounds.

Clade 2 (purple) contains six tomato MATEs in addition to proteins known to mediate the transport of proanthocyanidins or anthocyanins to the vacuole, such as TRANSPARENT TESTA 12 (TT12/DTX41) from Arabidopsis [28, 47], BrTT12 from turnip [50], MtMATE1 from *Medicago truncatula* [27], MdMATE1 and MdMATE2 from apple [51] as well as VvMATE1 and VvAnthoMATE from grapevine [29, 52]. Among the tomato MATE proteins present in this group, Solyc12g006360 seems to be the best candidate for vacuolar sequestration of anthocyanins due to its close relation with characterized transporters. This result is significant, given the recent interest in breeding vegetables for high-nutrient density [11]. Also in this clade is SbMATE2 from sorghum, which transports toxic hydroxynitrile glucosides (e.g., dhurrin) to the vacuole of cells in diverse tissues of the plant [53].

Clade 3 (green) contains 21 tomato MATEs along with only three from other species that have been functionally characterized. NtJAT1 from tobacco is a jasmonate-inducible alkaloid carrier expressed in stem, roots, and leaves [54]. In coordination with NtJAT2, NtJAT1 plays a role in the vacuolar sequestration of alkaloids in tobacco (e.g., nicotine, anabasin, hyoscyamine, and berberine) [44, 54]. Interestingly, Solyc02g091070 and Solyc02g091080 show a close phylogenetic relationship to NtJAT1, indicating they may also transport alkaloids in tomato. In Arabidopsis, ABERRANT LATERAL ROOT FORMATION 5 (ALF5/DTX19) is expressed in root epidermal cells and necessary for protecting roots from toxic compounds in the soil [55]. AtDTX18 is responsible for the secretion of the coumaroylagmatine and other hydroxycinnamic acid amides in response to *Phytophthora infestans* colonization [56]. From the few genes functionally characterized in the group, it is possible that they transport toxic compounds to specific parts of the plant as a component of plant defense mechanisms.

Clade 4 (brown) consists of eleven tomato MATEs and six functionally characterized transporters from other species. In Arabidopsis, ADS5/DTX47 participates in plant immune response by transporting salicylic acid (SA) upon induction by biotic stress [24]. On the other hand, ACTIVATED DISEASE SUSCEPTABILITY 1 (ADS1/ADP1/DTX51) inhibits SA accumulation [23]. The Golgi-localized BUSH AND CHLOROTIC DWARF 1 (BCD1/ZRIZ1/ZRZ/ABS4/ZF14/DTX48) is expressed in flowers, shoots, and the hypocotyl. It plays a role in Fe homeostasis, including during organ initiation and development [57, 58]. MtMATE55 was experimentally confirmed to play a similar role in the model legume, *Medicago truncatula* [22] further supporting the usefulness of phylogeny to predict function in the MATE family even in distantly related plant species. ABNORMAL SHOOT 3-LIKE 1 (ABS3L1/DTX50) and ABS3L2/DTX52 are both implicated in the inhibition of hypocotyl cell elongation [59]. RESISTANT TO HIGH CO_2_ 1 (RHC1/DTX56) is localized to the plasma membrane and participates in responses to increased CO_2_ and stomatal closure by repressing HIGH LEAF TEMPERATURE 1 (HT1) and OPEN STOMATA 1 (OST1) protein kinases, thus linking this MATE transporter to CO_2_ signaling through bicarbonate sensing [60]. Overall, the MATE transporters in this group are related to Fe homeostasis and the transport of signaling molecules involved in diverse mechanisms of plant defense, growth, and development.

At last, clade 5 (pink) contains five tomato MATEs. The functionally characterize members of this group mediate citrate efflux and participate either in Fe uptake and metal homeostasis or Al^3+^ tolerance mechanisms. FERRIC REDUCTASE DEFECTIVE 3 (FRD3/DTX43) is essential for Zn tolerance in Arabidopsis by regulating Fe homeostasis [61]. Likewise, GmFRD3a and GmFRD3b are induced by Fe deficiency in soybean [62]. In proteoid roots of the legume *Lupinus albus*, LaMATE is induced under P deficiency conditions [63]. Importantly, functional analysis of MATE transporters of this group in Arabidopsis and cereals led to the development of useful genetic markers for improved crop tolerance to Al^3+^ in acidic soils [64]. Members of this clade have also been functionally characterized as citrate effluxers in several cereals, such as wheat (TaMATE1B) [65], rye (ScFRDL1 and ScFRDL2) [66], barley (HvAACT1, the ALUMINUM-ACTIVATED CITRATE TRANSPORTER 1) [67], rice (OsFDL1 and OsFRDL4) [68], sorghum (SbMATE) [64, 69], and maize (ZmMATE1) [70], not to mention in dicot species, such as cabbage (BoMATE) [19], eucalyptus (EcMATE1) [71], and the MATEs from legume models, LjMATE1 from *Lotus japonicus* [72] and MtMATE66 and MtMATE69 from *Medicago truncatula* [22]. The three tomato transporters in the subgroup (Solyc01g008420, Solyc01g087150, Solyc11g065820) may be significant to improve tolerance to acidic soils in Solanaceae crops.

Also, a clear subclade containing two MATEs from tomato and three from Arabidopsis can be noticed at the root of clade 5. The ENHANCED DISEASE SUSCEPTIBILITY (EDS5/SCORD3/SID1/DTX47) from Arabidopsis participates in SA signaling for disease resistance [73]. Surprisingly, EDS5 localizes to the chloroplast envelope of epidermal cells [32] and mediates SA influx from this organelle to the cytosol upon stress [31]. It is quite significant that this small subclade displays a contrasting role in transport direction (as an influxer) along with a distinct subcellular localization. Given that Solanaceae crops are often vulnerable to multiple diseases, these two tomato MATE genes may be key to breeding more disease-tolerant varieties and are worthy of functional studies.

### Tandem gene duplications and synteny of MATE transporters in the tomato genome

The expansion of the MATE family in plant genomes in relation to other kingdoms is quite remarkable [14, 74]. This may relate to the sessile lifestyle of plants, which calls for many defense metabolites, and hence, membrane transporters to carry out efflux or vacuolar sequestration of toxic substances in order to cope with all the biotic and abiotic stresses inherent to the environment [75]. Furthermore, in general, the presence of multiple paralogs in multigene families may also relate to the recurring polyploidization events of the angiosperm lineage, which generated gene duplicates that have often been retained in extant plant genomes [76]. Over time, these duplicates may have culminated in sub- or neofunctionalization, and subsequently, acquired new functions that are occasionally retained, thus resulting in functional diversity and proliferation of genes derived from a common ancestor gene [77]. The identification of closely related paralogs in genomes is useful to discover potential gene redundancies, whereas identifying true orthologues between species can lead to the creation of hypotheses of common gene functions in other species.

In order to establish strong evidence of homology, we assessed the microsynteny within the tomato genome, as well as the syntenic block conservation between the tomato and Arabidopsis genomes. Our analysis revealed 13 tandem duplication segments containing 33 MATE genes on 6 chromosomes (Additional file 3 Table S3). Therefore, in-tandem MATE duplicates comprise 55% of the gene family in the tomato genome, which supports the role of this evolutionary mechanism in the expansion of the gene family [15, 78]. In plants, the degree of paralog fractionation is usually more related to the functional category than the genetic proximity between species. That is, genes linked to metabolic functions tend to be present in fewer copies in relation to those involved in regulation and stimulus responses [79], such as MATE genes. In addition, tandem duplications may result in an intensification of gene expression. This fact has been observed in corn, which varieties with three identical, in-tandem MATE genes showed greater tolerance to Al^3+^ toxicity due to an increased overall expression of these genes [80].

**Fig. 2:**
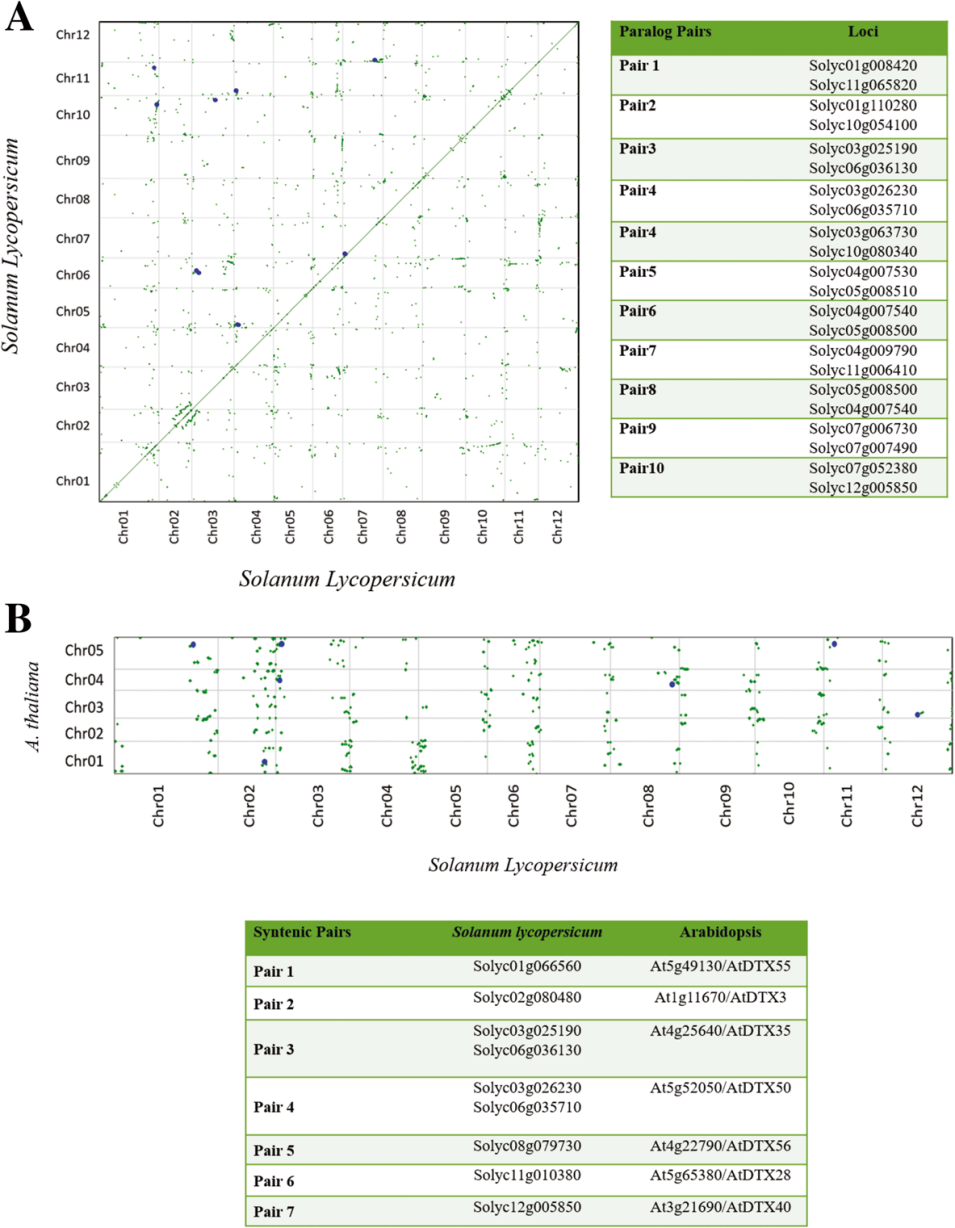
Syntenic analyses of MATE genes in the tomato genome. **a** Identification of paralog pairs in microsyntenic blocks within the tomato genome. Ten gene pairs were identified. **b** Synteny analysis between MATE transporters in the tomato and *Arabidopsis thaliana* genomes. Seven syntenic paralogs were found in this analysis. Blue dots were plotted according to gene coordinates within the respective chromosomes

Ten pairs of syntenic MATE paralogs were found within the tomato genome **(**Fig. 2a **),** whereas seven ortholog pairs were identified in syntenic blocks between tomato and Arabidopsis (Fig. 2b). In the phylogenetic context, the tomato paralogs belong to clade 1 (two syntenic pairs), as well as clades 2 and 4 (three pairs each), thus establishing strong evidence of common ancestry (i.e., orthology) as well as allowing us to propose robust hypotheses of functional conservation between the MATE genes that have already been functionally characterized in Arabidopsis and their syntenic pairs in tomato, especially when the expression patterns are preserved over the course of evolution.

We also observed that, in some instances, duplicated tomato MATE genes showed conserved synteny with Arabidopsis genes (Fig. 2b). Solyc03g025190/MTP77 is microsyntenic to Solyc06g036130 (paralog pair 3), and syntenic to At4g25640/AtDTX35/FFT/ (ortholog pair 3, Fig. 2b). Based on this information, both of these tomato transporters might function as vacuolar chloride channels related to regulation of cell turgescence (clade 1, Fig. 1). Solyc03g026230 is microsyntenic to Solyc06g035710 (paralog pair 4), and orthologous to At5g52050/AtDTX50/ABS3L2 (ortholog pair 4). AtDTX50 (clade 4) is implicated in plant development and growth by potentially inhibiting hypocotyl elongation, although its substrate remains unknown [59]. Solyc12g005850 is microsyntenic to Solyc07g052380 (paralog pair 10), and syntenic to At3g21690/AtDTX40 (ortholog pair 7). They belong to clade 2 and are probably connected to the transport of anthocyanins or other flavonoids, since At3g59030/TT12/DTX41 [47], BrTT12 [50], VvAM1[48], and MtMATE1 [27] cluster together in this clade. At last, we noticed that the Solyc02g080480/Solyc02g080490 in-tandem pair duplicate on chromosome 2 is syntenic to At1g11670/DTX36 (ortholog pair 2) and, given their location in clade 2, they are probably connected to the transport of anthocyanins or other flavonoids.

Therefore, we identified tomato MATE transporters potentially transporting flavonoids, alkaloids, and signaling molecules. Likely, their physiological functions have been conserved at least since the last common ancestor between these two species, which is estimated to have existed circa 150 million years ago [81].

### Expression patterns of MATE genes in the tomato plant

**Fig. 3:**
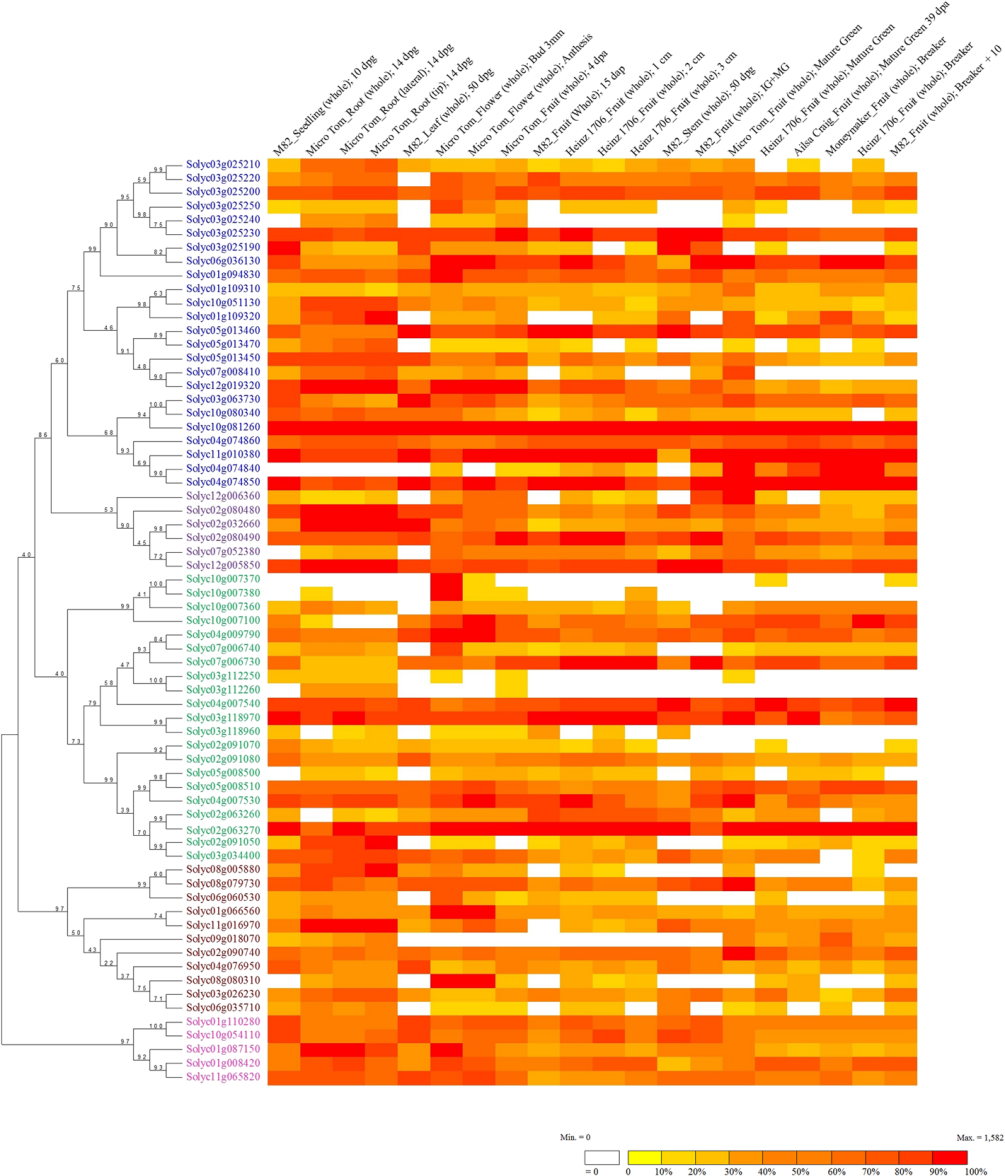
Expression profiling of tomato MATE transporters. Selected RNA-Seq samples were analyzed using the *TomExpress* platform. Genes are ordered according to their phylogenetic relationships

The expression analysis of the 67 tomato MATE genes identified was performed using the *TomExpress* platform (http://gbf.toulouse.inra.fr/tomexpress/www/query.php). A heatmap of gene expression was generated with 19 representative samples in different organs. The genes are displayed according to their phylogenetic associations (Fig. 3). All genes in this family are expressed. While 33 tomato MATE genes are constitutively expressed in the dataset, 34 showed changes in their transcriptional activity.

Clade 1 (blue) contains tree genes with constitutive, high expression (Solyc10g081260; Solyc11g010380 and Solyc04g074850). Solyc03g025240 has the lowest and most specific expression in the group, which, given its close phylogenetic relationship with NtJAT2 from tobacco (Fig. 1), supports a role of transporting alkaloids in roots, flowers, and fruits in a very early developmental stage. A previous attempt to assign a function for Solyc10g081260 as a potential phenolics transporter took into account a < 2-fold change of expression in fruit tissues between cv. M82 and its near-isogenic introgression line (IL7–3 from *S. pennellii*) [8]. Importantly, the study did not consider the ubiquitous, constitutive and high expression throughout the plant. Although our studies do not rule out this possibility, we propose that Solyc10g081260 is likely ought to transport alkaloids.

In contrast, only two tomato MATEs in clade 2 (purple) showed constitutive expression (Solyc02g080490 and Solyc12g005850). Other members of this clade show varied levels of transcriptional activity in diverse tissues.

In relation to the movement of defense compounds, clade 3 members (green) encompass Solyc02g063270, which is highly expressed in all tissues studied. On the other hand, two members (Solyc03g11250, and Solyc03g112260) showed varying, low expression across tissues, while Solyc10g007380 was highly expressed in flowers, and some expression during the early stages of fruit development.

Of the eleven transporters in clade 4 (brown), four showed constitutive expression, although at mid-to-low levels in most tissues analyzed. Given the close phylogenetic relationship (Fig. 1) and similar expression pattern (Fig. 3), it is tempting to suggest that Solyc08g080310 plays the same role of that assigned for the Arabidopsis BCD1/DTX48, which is expressed in flowers and vegetative shoots and is involved in Fe nutrition during organ initiation and development [15, 57, 58].

Unlike other clades, all MATE genes in clade 5 (pink) were constitutively expressed, with varying transcriptional intensities. Members of this group have been related to the transport of citrate and detoxification of Al^3+^ in roots as well as Fe translocation throughout the plant. The constitutive expression patterns of clade 5 members suggest they participate in physiological mechanisms throughout the whole plant.

### Regulatory gene networks involving MATE transporters and transcriptional master regulators

**Fig. 4:**
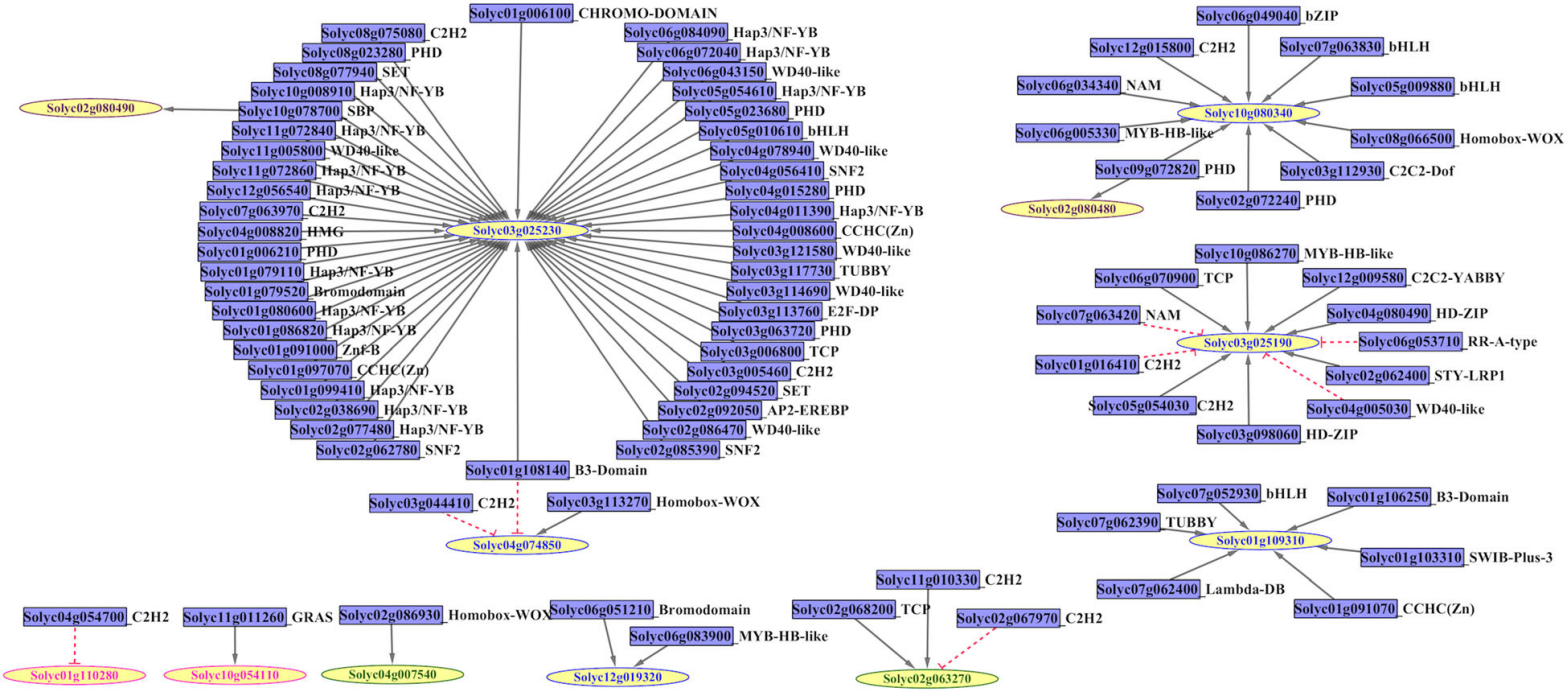
Regulatory gene networks involving transcription master regulators and MATE transporter genes. **a** A stringent threshold (ρ ≥ |0.8|) was set and visualization was produced in Cytoscape. Nodes for MATE genes are represented by yellow ellipses (the color of font and contour used represents their respective phylogenetic clade as shown in Figs. 1 and 3). Nodes for transcriptional regulators are represented by blue rectangles. Given the respective putative function of the gene products, the directionality of each interaction was assumed to occur from the putative regulator to the MATE gene. Positive interactions are indicated by gray edges with arrowheads and negative interactions by dashed edges with T ends. The family of each transcriptional regulator is indicated

In all living cells, the major regulation point of gene expression lies within the confines of transcriptional initiation. Transcription factors, ancillary transcription regulators (which act by interacting with transcription factors due to the lack of a DNA-binding motif), and chromatin regulators (remodelers and modifiers) are master regulators of gene expression. Therefore, gene networks for all transcriptionally modulated genes in a genome are expected to be centered on these master regulators of transcription. PlantTFcat is a useful analysis tool to identify proteins with signature domains specific to 108 families of master transcription regulators [82]. First, we assessed the tomato genome for these regulators and found 3992 genes encoding conserved domains of characterized proteins implicated in transcriptional regulation (11.4% of the ITAG3.10 proteome; Additional file 4 Table S4). We then used *TomExpress* to assess the expression data of 3169 regulatory genes available in the platform along with the 67 tomato MATE genes previously found (Additional file 5 Table S5). The non-parametric Spearman’s rank-order correlation was chosen for our co-expression analysis over more complex calculations due to its robustness to generate biologically relevant gene networks [83]. Additional file 5 Table S5 shows the matrix of all correlation values for expression values between each pair of regulatory and MATE genes from a set of 174 biological samples. At the conservative threshold of ρ ≥ |0.8|, only 78 positive and 8 negative correlations involving 12 MATE genes were found (Fig. 4). Interestingly, the constitutive MATE transporter Solyc03g025230 was positively linked to 46 transcriptional regulators, while Solyc03g025190 and Solyc10g080340 were associated with 11 and 10 transcriptional regulators, respectively. Alone, genomic location (i.e., the local chromatin structure) cannot explain the gene associations found for Solyc03g025230, since this gene belongs to a large in-tandem cluster containing six MATE genes, each with a distinct expression profile (Table 1, Fig. 3). Another case worth mentioning is the 7 positive and 4 negative associations for Solyc03g025190 (MTP77). As mentioned above, this MATE was previously reported to be induced by the MYB transcription factor ANT1/MYB113 (Solyc10g086260) [30]. However, our results rather revealed a strong association (ρ=0.827) with its in-tandem duplicate, MYB28 (Solyc10g086270), while the MTP77 transcription pattern was more weakly correlated (ρ=0.644) with the MYB113 expression profile (Fig. 4). In light of current genomic data, this incongruence calls for an experimental revalidation of the data reported in [30] for establishing Solyc10g086260 as the transcription factor responsible for the high anthocyanin phenotype of the *ant1* mutant through induction of MYB expression.

**Table 1:**
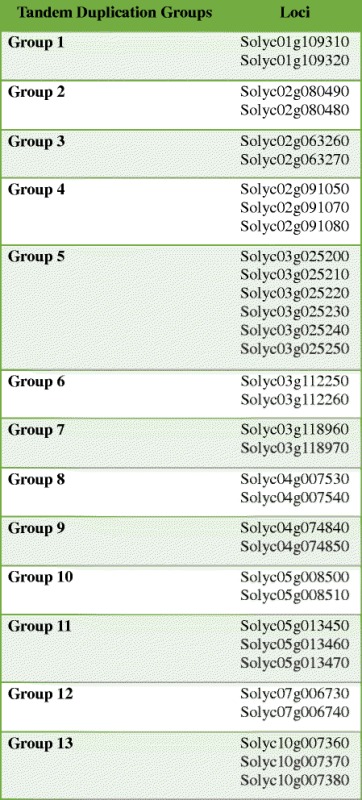
In-tandem MATE gene duplicates in the *S. lycopersicum* genome

The primary Arabidopsis transcription factor recognized to induce the expression of a MATE malate effluxer (At1g51340/DTX42) in roots exposed to Al^3+^ toxicity is the C_2_H_2_/C_2_HC zinc-finger protein SENSITIVE TO PROTON RHIZOTOXICITY 1 (STOP1) [21]. A potential homolog of STOP1 in tomato is Solyc11g017140, although no strong associations were found between this transcription factor and any tomato MATE gene. It would be interesting to further verify tolerance to acidic soils regarding expression of Solyc11g017140 and clade 5 MATE genes.

A few transcription factors were strongly linked to more than one MATE, such as the SQUAMOSA BINDING PROTEIN (SBP) transcription factor Solyc10g078700 (SlySBP15), which was positively associated with Solyc03g025230 and Solyc02g080490. The B3-domain factor Solyc01g108140 was positively associated with Solyc03g025230 but negatively linked to Soly04g074850. These regulators have not been functionally characterized yet, but it will be interesting to understanding the general impact of their loss of function on gene expression of their associated MATE genes. Although the initial correlation threshold used here may be overly stringent, since a higher number of biologically relevant links are expected to exist especially for the 34 non-constitutive MATE genes, the strong MATE-regulator associations revealed here will help address important questions to discover novel regulators of MATE gene expression.

### Assessing tomato MATE transporters in the genome context of metabolic gene clusters

Metabolic gene clusters can be defined as a set of at least three structural genes related to a particular biosynthetic pathway that are grouped in a defined region (not necessarily consecutive, but immediate) of the genome of an organism. Bacteria and fungi are well known to contain metabolic gene clusters, but plants have been regarded for a long time to be devoid of them. Nevertheless, co-expressed metabolic gene clusters have started being noticed in the genomes of some plant species, particularly for terpenoids [84, 85] and alkaloids [86], but remarkably not for carotenoids or phenolic compounds [87]. In tomato, several metabolic gene clusters have been found, such as one for the steroidal glycoalkaloid α-solanine (10 genes on two blocks located on chromosomes 7 and 12) [86] and several for terpenoid biosynthesis on chromosomes 1, 2, 6, 8 and 10 [10]. Remarkably, in other species, two MATE transporters were found within metabolic gene clusters. In sorghum, SbMATE2 belongs to a metabolic gene cluster [87]. that groups structural genes coding related to dhurrin biosynthesis [53]. Another such case is for the biosynthesis of monoterpene indol alkaloids (e.g., vinblastine, vincristine, catharantine) in *Catharanthus roseus* [88]. In both instances, there is an expectation that they play a function in the metabolism of the respective compound. This hypothesis was confirmed for SbMATE2 but it remains missing for the *Catharanthus* MATE transporter.

We assessed the locations of all MATE transporter genes against metabolic gene clusters in the tomato genome. The closest gene to a known metabolic gene cluster is Solyc08g005880 which lies 73.7 Kb downstream the tail of metabolic gene cluster, an intervening genomic region containing six unrelated genes. We also examined expression correlation values for each of the genes in the metabolic cluster against this MATE transporter gene, and found no strong evidence of co-expression (ρ≥0.8; Additional file 6 Figure S1). Therefore, our analyzes showed no evidence of MATEs belonging to metabolic gene clusters in tomato.

## Conclusions

Overall, the global analysis of 67 MATE genes identified in the tomato genome revealed potential functional relationships with transporters characterized in other plant species, as well as potentially interesting targets for functional studies. Such analyses are crucial to identify key genes for breeding purposes in tomato as well as to better understand specialized metabolism in the Solanaceae as a whole.

## Methods

### Identification of MATE transporters in the tomato genome

The full tomato protein dataset (ITAG v.2.4 release: ftp://ftp.solgenomics.net/tomato_genome/annotation/ITAG2.4_release/ITAG2.4_proteins.fasta) was submitted to the TransportTP transporter prediction tool (http://bioinfo3.noble.org/transporter/) [42] for identification and classification of membrane transporters into transporter families according to the Transporter Classification system (TCDB, http://www.tcdb.org) [89]. Manual curation for the MATE family followed as previously described [41]. Briefly, transporters were assessed against the expected features of the MATE transporters in plants (e.g., 12 transmembrane domains, ~500 amino acid residues, high similarity to plant MATEs in curated as well as comprehensive databases), and assigned a functionality confidence level (1 for highest confidence the gene product is functional; 2 for a likely functional gene product, with a few features off or missing; and 3 for possibly a pseudogene or non-functional MATE transporter).

### Phylogenetic analyses

The evolutionary analysis was conducted in MEGA7 [90] and involved 156 full-length MATE amino acid sequences from tomato (67 sequences), Arabidopsis (56 sequences, obtained through a de novo genomic analysis of the Arabidopsis genome v. TAIR10 with the TransportTP tool, as described above) and those functionally characterized from other plant species (33 sequences, from our own comprehensive literature mining). The phylogenetic analyses were inferred using MEGA 6 (www.megasoftware.net) by the Maximum Likelihood method with a bootstrap of 1000 replicates, based on the JTT matrix-based model [91]. The initial trees for the heuristic search were obtained automatically by applying Neighbor-Join and BioNJ algorithms to a matrix of pairwise distances estimated with the JTT model. The topology was selected with superior log-likelihood value. The tree was drawn to scale, with branch lengths measured in number of substitutions per site.

### Analyses of synteny

The CoGe comparative genomic toolkit (https://genomevolution.org/coge/) [92] was used to identify in-tandem MATE duplications in tomato, syntenic genes within its genome, as well as syntenic (collinear) gene blocks between tomato and *Arabidopsis thaliana*. In-tandem gene duplicates in a genome (defined here as similar genes at most 10 genes apart with a threshold e-value ≤10e-4) were determined using Blast2raw script in the CoGe toolkit. Syntenic blocks (microsynteny within a genome or synteny between two genomes) were identified with SynMap thorugh the DAGchainer algorithm using the relative gene order option with a minimum of 5 gene pairs aligned and a maximum distance of 20 genes between two matches. QuotaAlign was used to merge adjacent syntenic blocks [93].

### Transcriptional profiling of MATE transporters

Relative expression of the 67 genes of tomato MATE family was carried out using the TomExpress platform (http://gbf.toulouse.inra.fr/tomexpress/www/query.php), as previously described [94]. This database contains public tomato RNA-Seq datasets for diverse experiments and serves as a hub for transcription data mining for the species. Normalized counts per gene derived from the “median ratio normalization” method [85] was used to generate the heatmap of relative expression.

### Identification and categorization of tomato transcriptional regulators in tomato

The ITAG3.1 tomato proteome sequence dataset (ftp://ftp.solgenomics.net/tomato_genome/annotation/ITAG3.1_release/ was loaded into the PlantTFcat analysis tool (http://plantgrn.noble.org/PlantTFcat/#; [82]) for identification and automatic classification of transcription factors, chromatin modifiers and other transcriptional regulators into protein families.

### Gene association analyses

Normalized RNA-Seq expression data (174 experiments) were obtained from the TomExpress platform [94]. Expression correlations between MATE and transcriptional regulators were established using Spearman’s rank correlation coefficient with a stringent threshold (ρ ≥ |0.80|) to predict potential gene regulatory networks [83]. The ρ correlation matrix was produced in Microsoft Excel for Mac (v.15) using the formula = CORREL(RANK.AVG(X,X,1), RANK.AVG (Y,Y,1)) where X and Y are strings of expression data (e.g., X_n_:X_n + 173_ and Y_n_:Y_n + 173_) for each gene pair under analysis (cf. Additional file 5 Table S5). Visualization of the network was created in Cytoscape v.3.5.1 [95].

## Additional files

Additional file 1: Table S1. Major gene product features of MATE family of membrane transporters identified in the *S. lycopersicum* genome. (DOCX 33 kb)

Additional file 2: Table S2. TransportTP output for the MATE gene family genes identified in the *S. lycopersicum* genome. (XLSX 25 kb)

Additional file 3: Table S3. In-tandem MATE gene duplicates in the *S. lycopersicum* genome. (DOCX 13 kb)

Additional file 4: Table S4. Identification and annotation of transcriptional regulators in tomato. (XLSX 1403 kb)

Additional file 5: Table S5. Gene expression and Spearman’s rank correlations between MATE transporters and putative transcriptional regulators identified in tomato. (XLSX 8715 kb)

Additional file 6: Figure S1. Co-expression analysis of genes in the terpene metabolic gene cluster and proximal MATE transporter on chromosome 8 of tomato. Numbers in columns designate the genes numbered in rows. Cells containing correlation values were shaded using a color scale from blue (for negative correlations) to red (positive correlations). (PDF 35 kb)

## Abbreviations

ABA: Abscisic acid
CoGe: Comparative Genomics
DTX: Detoxification proteins (=MATE transporters)
IL: Introgression line
IPR: InterPro domain
ITAG: International Tomato Annotation Group
JTT: Jones-Taylor-Thornton
MATE: Multidrug and Toxic Compound Extrusion; Multi-Antimicrobial Extrusion
MEGA: Molecular Evolutionary Genetic Analysis
PF: Pfam domain
SA: Salicylic acid
TAIR: The Arabidopsis Information Resource
TCDB: Transporter Classification Database
TMD: Transmembrane domains
