# Supplementary material for: Global analysis of the MATE gene family of metabolite transporters in tomato
Source: BMC Plant Biol. 2017 Oct 30;17:185. doi: 10.1186/s12870-017-1115-2 (PMC5663081; doi:10.1186/s12870-017-1115-2)
Supplement: Supplementary file 1 — Major gene product features of MATE family of membrane transporters identified in the S. lycopersicum genome. (DOCX 33 kb) [file 12870_2017_1115_MOESM1_ESM.docx]

**Supplemental Table S1.** Members of the MATE family of membrane transporters (TCDB 2.A.66.1) in the *S. lycopersicum* genome.

| Locus | Phylogenetic Clade | Protein size | Transmembrane domains  (TMHMM) | Best BLAST match (TCDB database) | species | Evalue* | TransportTP Confidence | Manual curation of classification  (confidence level) ** |
| --- | --- | --- | --- | --- | --- | --- | --- | --- |
| Solyc01g109310 | **1** | 476 aa | 12 | 2.A.66.1.44 - TT12 | cotton | 2.7e-118 | 100 | 1 |
| Solyc01g109320 | **1** | 497 aa | 12 | 2.A.66.1.44 - TT12 | cotton | 1.0e-127 | 100 | 1 |
| Solyc01g094830 | **1** | 1245 aa | 12 | 2.A.66.1.44 - TT12 | cotton | 9.5e-115 | 100 | 1 |
| Solyc03g025200 | **1** | 475 aa | 12 | 2.A.66.1.44 - TT12 | cotton | 2.0e-114 | 100 | 1 |
| Solyc03g025210 | **1** | 494 aa | 12 | 2.A.66.1.44 - TT12 | cotton | 6.1e-112 | 100 | 1 |
| Solyc03g025240 | **1** | 399 aa | 12 | 2.A.66.1.44 - TT12 | cotton | 2.1e-106 | 100 | 1 |
| Solyc03g063730 | **1** | 413 aa | 12 | 2.A.66.1.44 - TT12 | cotton | 7.1e-112 | 100 | 1 |
| Solyc03g025190 | **1** | 506 aa | 12 | 2.A.66.1.44 - TT12 | cotton | 3.1e-132 | 100 | 1 |
| Solyc03g025220 | **1** | 505 aa | 9 | 2.A.66.1.44 - TT12 | cotton | 3.1e-132 | 90.9 | 1 |
| Solyc03g025230 | **1** | 500 aa | 12 | 2.A.66.1.44 - TT12 | cotton | 8.7e-124 | 100 | 1 |
| Solyc03g025250 | **1** | 501 aa | 11 | 2.A.66.1.44 - TT12 | cotton | 8.3e-113 | 100 | 1 |
| Solyc04g074840 | **1** | 503 aa | 12 | 2.A.66.1.44 - TT12 | cotton | 4.3e-125 | 100 | 1 |
| Solyc04g074850 | **1** | 482 aa | 12 | 2.A.66.1.44 - TT12 | cotton | 6.8e-124 | 100 | 1 |
| Solyc04g074860 | **1** | 569 aa | 13 | 2.A.66.1.44 - TT12 | cotton | 4.4e-103 | 100 | 1 |
| Solyc05g013450 | **1** | 515 aa | 13 | 2.A.66.1.44 - TT12 | cotton | 2.0e-144 | 100 | 1 |
| Solyc05g013460 | **1** | 514 aa | 12 | 2.A.66.1.44 - TT12 | cotton | 1.1e-140 | 100 | 1 |
| Solyc05g013470 | **1** | 504 aa | 12 | 2.A.66.1.44 - TT12 | cotton | 3.6e-142 | 100 | 1 |
| Solyc06g036130 | **1** | 504 aa | 12 | 2.A.66.1.44 - TT12 | cotton | 4.1e-132 | 100 | 1 |
| Solyc07g008410 | **1** | 480 aa | 11 | 2.A.66.1.44 - TT12 | cotton | 2.0e-137 | 100 | 1 |
| Solyc10g051130 | **1** | 478 aa | 12 | 2.A.66.1.44 - TT12 | cotton | 3.2e-117 | 100 | 1 |
| Solyc10g080340 | **1** | 507 aa | 12 | 2.A.66.1.44 - TT12 | cotton | 1.2e-122 | 100 | 1 |
| Solyc10g081260 | **1** | 485 aa | 11 | 2.A.66.1.44 - TT12 | cotton | 8.5e-113 | 100 | 1 |
| Solyc11g010380 | **1** | 493 aa | 11 | 2.A.66.1.44 - TT12 | cotton | 3.9e-126 | 100 | 1 |
| Solyc12g019320 | **1** | 502 aa | 12 | 2.A.66.1.44 - TT12 | cotton | 5.4e-135 | 100 | 1 |
| Solyc02g032660 | **2** | 504 aa | 12 | 2.A.66.1.44 - TT12 | cotton | 2.0e-161 | 100 | 1 |
| Solyc02g080480 | **2** | 499 aa | 12 | 2.A.66.1.44 - TT12 | cotton | 6.1e-159 | 100 | 1 |
| Solyc02g080490 | **2** | 504 aa | 12 | 2.A.66.1.44 - TT12 | cotton | 6.6e-157 | 100 | 1 |
| Solyc07g052380 | **2** | 503 aa | 12 | 2.A.66.1.44 - TT12 | cotton | 1.7e-162 | 100 | 1 |
| Solyc12g005850 | **2** | 512 aa | 12 | 2.A.66.1.44 - TT12 | cotton | 1.0e-165 | 100 | 1 |
| Solyc12g006360 | **2** | 503 aa | 12 | 2.A.66.1.44 - TT12 | cotton | 0.0 | 100 | 1 |
| Solyc02g091050 | **3** | 474 aa | 12 | 2.A.66.1.19 - putative MATE transporter | tobacco | 0.0 | 100 | 1 |
| Solyc02g091070 | **3** | 471 aa | 12 | 2.A.66.1.19 - putative MATE transporter | tobacco | 0.0 | 100 | 1 |
| Solyc02g091080 | **3** | 507 aa | 12 | 2.A.66.1.19 - putative MATE transporter | tobacco | 0.0 | 100 | 1 |
| Solyc02g063260 | **3** | 492 aa | 12 | 2.A.66.1.19 - putative MATE transporter | tobacco | 0.0 | 100 | 1 |
| Solyc02g063270 | **3** | 454 aa | 12 | 2.A.66.1.19 - putative MATE transporter | tobacco | 0.0 | 100 | 1 |
| Solyc03g034400 | **3** | 470 aa | 12 | 2.A.66.1.19 - putative MATE transporter | tobacco | 0.0 | 100 | 1 |
| Solyc03g112250 | **3** | 489 aa | 12 | 2.A.66.1.19 - putative MATE transporter | tobacco | 6.8e-136 | 100 | 1 |
| Solyc03g112260 | **3** | 519 aa | 12 | 2.A.66.1.19 - putative MATE transporter | tobacco | 7.2e-126 | 100 | 1 |
| Solyc03g118960 | **3** | 419 aa | 8 | 2.A.66.1.8 - At2g04040 | Arabidopsis | 2.7e-54 | 100 | 2 |
| Solyc03g118970 | **3** | 495 aa | 12 | 2.A.66.1.19 - putative MATE transporter | tobacco | 2.3e-141 | 100 | 1 |
| Solyc04g007530 | **3** | 481 aa | 12 | 2.A.66.1.19 - putative MATE transporter | tobacco | 0.0 | 100 | 1 |
| Solyc04g007540 | **3** | 480 aa | 12 | 2.A.66.1.19 - putative MATE transporter | tobacco | 6.3e-137 | 100 | 1 |
| Solyc04g009790 | **3** | 1255 aa | 13 | 2.A.66.1.19 - putative MATE transporter | tobacco | 2.2e-127 | 100 | 1 |
| Solyc05g008500 | **3** | 456 aa | 11 | 2.A.66.1.19 - putative MATE transporter | tobacco | 4.0e-151 | 100 | 1 |
| Solyc05g008510 | **3** | 478 aa | 12 | 2.A.66.1.19 - putative MATE transporter | tobacco | 0.0 | 100 | 1 |
| Solyc07g006730 | **3** | 489 aa | 12 | 2.A.66.1.8 - At2g04040 | Arabidopsis | 3.3e-135 | 100 | 1 |
| Solyc07g006740 | **3** | 471 aa | 12 | 2.A.66.1.19 - putative MATE transporter | tobacco | 3.7e-127 | 100 | 1 |
| Solyc10g007100 | **3** | 488 aa | 11 | 2.A.66.1.8 - At2g04040 | Arabidopsis | 0.0 | 100 | 1 |
| Solyc10g007360 | **3** | 482 aa | 12 | 2.A.66.1.6 - ALF5 | Arabidopsis | 0.0 | 100 | 1 |
| Solyc10g007370 | **3** | 547 aa | 12 | 2.A.66.1.6 - ALF5 | Arabidopsis | 3.9e-175 | 100 | 1 |
| Solyc10g007380 | **3** | 512 aa | 12 | 2.A.66.1.6 - ALF5 | Arabidopsis | 2.0e-176 | 100 | 1 |
| Solyc01g066560 | **4** | 495 aa | 12 | 2.A.66.1.19 - putative MATE transporter | tobacco | 6.2e-67 | 100 | 1 |
| Solyc02g090740 | **4** | 505 aa | 12 | 2.A.66.1.19 - putative MATE transporter | tobacco | 5.7e-77 | 100 | 1 |
| Solyc03g026230 | **4** | 527 aa | 11 | 2.A.66.1.19 - putative MATE transporter | tobacco | 1.2e-84 | 100 | 1 |
| Solyc04g076950 | **4** | 550 aa | 12 | 2.A.66.1.19 - putative MATE transporter | tobacco | 5.9e-79 | 100 | 1 |
| Solyc06g035710 | **4** | 520 aa | 13 | 2.A.66.1.19 - putative MATE transporter | tobacco | 1.2e-86 | 90.9 | 1 |
| Solyc06g060530 | **4** | 487 aa | 12 | 2.A.66.1.19 - putative MATE transporter | tobacco | 4.9e-70 | 100 | 1 |
| Solyc08g005880 | **4** | 534 aa | 13 | 2.A.66.1.44 - TT12 | cotton | 8.9e-121 | 100 | 1 |
| Solyc08g079730 | **4** | 480 aa | 12 | 2.A.66.1.19 - putative MATE transporter | tobacco | 7.6e-78 | 100 | 1 |
| Solyc08g080310 | **4** | 494 aa | 12 | 2.A.66.1.19 - putative MATE transporter | tobacco | 2.0e-88 | 100 | 1 |
| Solyc09g018070 | **4** | 466 aa | 12 | 2.A.66.1.19 - putative MATE transporter | tobacco | 1.0e-71 | 100 | 1 |
| Solyc11g016970 | **4** | 472 aa | 11 | 2.A.66.1.19 - putative MATE transporter | tobacco | 5.5e-74 | 100 | 1 |
| Solyc01g008420 | **5** | 536 aa | 12 | 2.A.66.1.43 - MATE efflux protein | maize | 0.0 | 100 | 1 |
| Solyc01g087150 | **5** | 525 aa | 13 | 2.A.66.1.24 - FRD3 | Arabidopsis | 0.0 | 100 | 1 |
| Solyc01g110280 | **5** | 557 aa | 11 | 2.A.66.1.11 – EDS5/SID1 | Arabidopsis | 0.0 | 100 | 1 |
| Solyc10g054110 | **5** | 375 aa | 6 | 2.A.66.1.11 – EDS5/SID1 | Arabidopsis | 1.7e-136 | 100 | 2 |
| Solyc11g065820 | **5** | 510 aa | 12 | 2.A.66.1.43 - MATE efflux protein | maize | 0.0 | 81.8 | 1 |

*Evalue of Blast search on TCDB database.

**Manual curation of classification (confidence levels: 1=likely to be a functional protein; 2=minor red flags, but likely a functional protein). Supplemental Table 1 shows the full output of Ciport analysis.
