# Supplementary material for: Global analysis of the MATE gene family of metabolite transporters in tomato
Source: BMC Plant Biol. 2017 Oct 30;17:185. doi: 10.1186/s12870-017-1115-2 (PMC5663081; doi:10.1186/s12870-017-1115-2)
Supplement: Supplementary file 3 — In-tandem MATE gene duplicates in the S. lycopersicum genome. (DOCX 13 kb) [file 12870_2017_1115_MOESM3_ESM.docx]

**Supplemental Table S3.** In-tandem MATE gene duplicates in the *S. lycopersicum* genome

| **Tandem Duplication Groups** | **Loci** |
| --- | --- |
| **Group 1** | Solyc01g109310  Solyc01g109320 |
| **Group 2** | Solyc02g080490  Solyc02g080480 |
| **Group 3** | Solyc02g063260  Solyc02g063270 |
| **Group 4** | Solyc02g091050  Solyc02g091070  Solyc02g091080 |
| **Group 5** | Solyc03g025200  Solyc03g025210  Solyc03g025220  Solyc03g025230  Solyc03g025240  Solyc03g025250 |
| **Group 6** | Solyc03g112250  Solyc03g112260 |
| **Group 7** | Solyc03g118960  Solyc03g118970 |
| **Group 8** | Solyc04g007530  Solyc04g007540 |
| **Group 9** | Solyc04g074840  Solyc04g074850 |
| **Group 10** | Solyc05g008500  Solyc05g008510 |
| **Group 11** | Solyc05g013450  Solyc05g013460  Solyc05g013470 |
| **Group 12** | Solyc07g006730  Solyc07g006740 |
| **Group 13** | Solyc10g007360  Solyc10g007370  Solyc10g007380 |
