# Supplementary material for: Global analysis of the MATE gene family of metabolite transporters in tomato
Source: BMC Plant Biol. 2017 Oct 30;17:185. doi: 10.1186/s12870-017-1115-2 (PMC5663081; doi:10.1186/s12870-017-1115-2)
Supplement: Supplementary file 6 — Co-expression analysis of genes in the terpene metabolic gene cluster and proximal MATE transporter on chromosome 8 of tomato. Numbers in columns designate the genes numbered in rows. Cells containing correlation values were shaded using a color scale from blue (for negative correlations) to red (positive correlations). (PDF 35 kb) [file 12870_2017_1115_MOESM6_ESM.pdf]

	Gene	1	2	3	4	5	6	7	8	9	10	11	Gene annotation
**1**	Solyc08g005630	1											Long-chain-alcohol oxidase
**2**	Solyc08g005640	-0.190	1										Terpene synthase
**3**	Solyc08g005650	-0.183	0.579	1									Cytochrome P450
**4**	Solyc08g005660	-0.262	0.668	0.562	1								Alkyl transferase
**5**	Solyc08g005680	-0.107	0.698	0.452	0.685	1							Z,Z-farnesyl pyrophosphate synthase
**6**	Solyc08g005690	0.127	-0.251	0.085	0.008	0.047	1						Syntaxin 32 *(low quality annotation)*
**7**	Solyc08g005700	0.113	-0.362	-0.021	-0.127	-0.152	0.644	1					Trigalactosyldiacylglycerol
**8**	Solyc08g005720	-0.272	0.597	0.384	0.628	0.572	-0.057	-0.150	1				Terpene synthase 18
**9**	Solyc08g005750	-0.012	-0.073	-0.160	-0.077	-0.044	0.019	0.007	0.078	1			Benzoyltransferase
**10**	Solyc08g005760	0.497	-0.428	-0.325	-0.414	-0.401	0.086	0.154	-0.254	0.296	1		Alcohol acyl transferase
**11**	**Solyc08g005880**	-0.260	0.330	-0.103	0.385	0.508	0.021	-0.134	0.485	0.186	-0.255	1	**MATE transporter**

No expression data were available on TomExpress database for Solyc08g005665 (Terpene synthase), Solyc08g005725 (Cytochrome P450) or Solyc08g005755 (Benzyl alcohol O-benzoyltransferase)
